# Occurrence of Numerous Cerebral White Matter Hyperintensities in Trauma Patients With Cerebral Fat Embolism: A Systematic Review and Report of Two Cases

**DOI:** 10.7759/cureus.45450

**Published:** 2023-09-18

**Authors:** Gregory S Huang, C. Michael Dunham, Elisha A Chance

**Affiliations:** 1 Trauma, Critical Care, and General Surgery Services, St. Elizabeth Youngstown Hospital, Youngstown, USA; 2 Trauma and Neuroscience Research Department, St. Elizabeth Youngstown Hospital, Youngstown, USA

**Keywords:** starfield pattern, cerebral white matter hyperintensities, traumatic injury, long bone fracture, cerebral fat embolism syndrome

## Abstract

There has been little effort to identify an overall occurrence of numerous cerebral white matter hyperintensities (NCWMH) on relevant brain magnetic resonance imaging (MRI) sequences in postinjury cerebral fat embolism syndrome (CFES) patients. Also, quantification of pre-CFES cognitive status, degree of neurologic deterioration, and presence of a skeletal fracture with CFES is nominal.

The authors performed a PubMed search and identified 24 relevant manuscripts. Two case reports from the authors’ institution were also used. The presence of NCWMH was assessed by reviewing T2-weighted image (T2WI), diffusion-weighted image (DWI), fluid-attenuated inversion recovery (FLAIR) figures and captions, and by evaluating manuscript descriptions. When pre-CFES cognitive status was described, it was categorized as Glasgow Coma Scale (GCS) score = 14-15 (yes or no). When the degree of neurologic deterioration was noted with CFES, it was classified as coma or GCS ≤ 8 (yes or no). When skeletal fractures were itemized, they were categorized as yes or no.

The total number of CFES patients was 133 (literature search was 131 and two author-described case reports). Of the 131 patients with manuscript MRI figures or descriptive statements, 120 (91. 6%) had NCWMH. Of 63 patients with a delineation of the MRI sequence, NCWMH appeared on DWI in 24, on T2WI in 57, and on FLAIR in 10 patients. Pre-CFES cognitive status was GCS 14-15 in 93.5% (58/62) of the patients. The CFES neurologic deterioration was coma or GCS ≤ 8 in 52.5% (62/118) of the patients. A skeletal fracture was present in 99.0% (101/102) of the CFES patients.

The presence of NCWMH in trauma patients with hospital-acquired neurologic deterioration and the presence of a skeletal fracture is consistent with CFES.

## Introduction

Fat embolism syndrome was first reported by Zenker in 1862. It has been associated with displaced long bone fractures and includes the clinical symptoms of respiratory distress, petechial rash, and neurological symptoms [1]. Many patients do not develop the classic triad of symptoms [2]. Cerebral fat embolism syndrome (CFES) is a rare and even fatal condition with a reported incidence in long bone fracture of 0.9% at the Harborview Trauma Center [3]. The National Hospital Discharge Survey found an incidence of 0.17% in patients with isolated or multiple orthopedic fractures across short-stay hospitals [4]. The neurological symptoms can be quite varied, making the diagnosis difficult [5,6]. Since CFES is a rare clinical entity, and neurologic deterioration can include coma and major motor deficits, securing the diagnosis is of paramount importance.

Magnetic resonance imaging (MRI) using T2-weighted image (T2WI), diffusion-weighted image (DWI), gadolinium contrast-enhanced images, and susceptibility-weighted imaging has improved the ability to make a diagnosis [5,7]. Parizel et al. first described a distinctive MRI pattern in identifying CFES as a “starfield pattern,” which are numerous cerebral white matter hyperintensities (NCWMH) that are seen on T2WI and DWI [7]. In the original description, these patterns of scattered bright spots were pathognomonic of acute cerebral microinfarcts [7]. Parizel noted that in the setting of CFES, DWI reveals the cytotoxic edema, which develops immediately, and on T2WI, the high signal abnormalities in gray and white matter reveal vasogenic edema, which takes longer to be evident [7]. A plethora of CFES MRI findings have been found and include hypointense microbleed and hyperintense ischemic signals involving various brain structures [5,6].

The authors of this article aimed to report two patient cases from their institution, a level I trauma center in northeast Ohio, USA, that developed CFES. Additionally, the authors performed a literature review to assess brain MRI findings in trauma patients with CFES but without a major neurologic deficit immediately following injury. We sought to determine the occurrence of NCWMH on DWI, T2WI, and/or fluid-attenuated inversion recovery (FLAIR) that implies the presence of ischemia, consistent with cerebral fat embolism. We hypothesized that this pattern would be seen in the majority of CFES cases. We also aimed to establish the pre-CFES cognitive status, degree of neurologic deterioration with CFES, and the presence or absence of a skeletal fracture with CFES.

The two cases herein were previously presented as an oral presentation at the Mercy Health Youngstown 2023 Regional Research Day on May 12, 2023.

## Case presentation

Methods

Literature Search

The authors performed a PRISMA (Preferred Reporting Items for Systematic Reviews and Meta-Analyses) systematic literature review. A PubMed literature search was performed on May 5, 2023, and implemented using all three words, “cerebral fat embolism,” in the citation title. The time period was restricted to the past five years.

**Figure 1:**
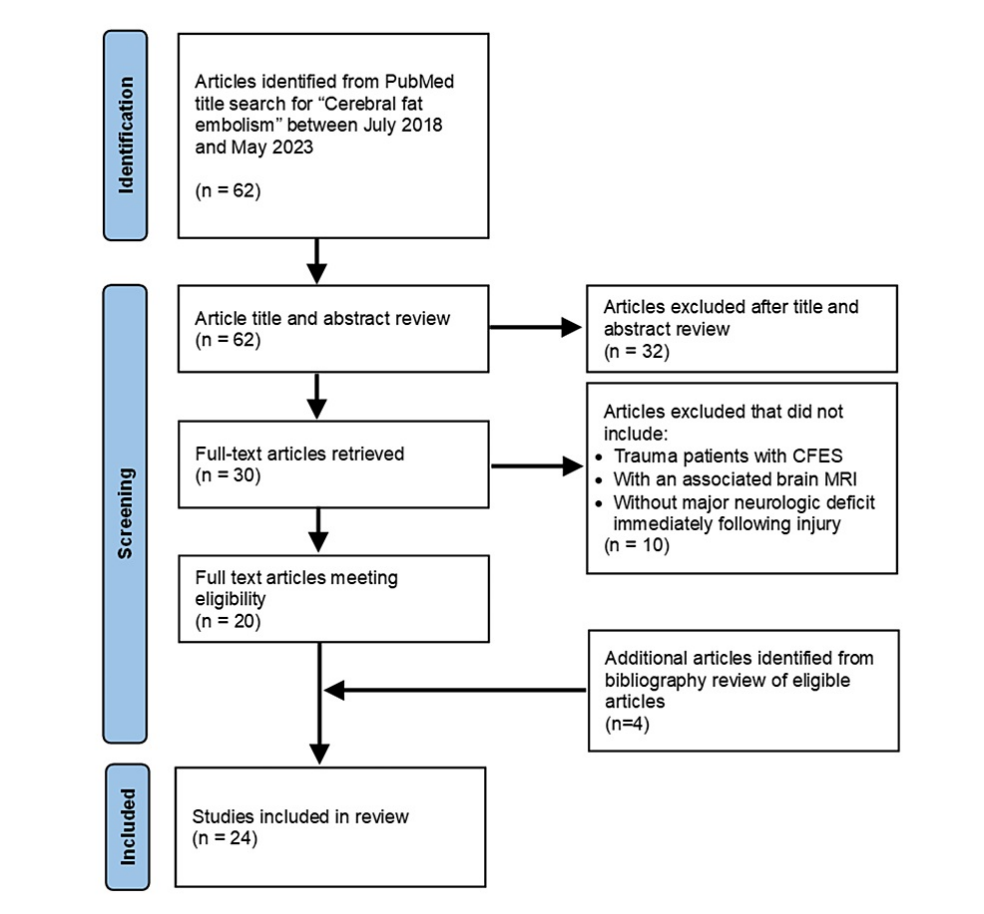
PRISMA flowchart of articles included in the systematic review PRISMA: Preferred Reporting Items for Systematic Reviews and Meta-Analyses.

The search yielded 62 publications between July 2018 and May 2023. Abstracts and titles were reviewed to determine if the described patient(s) had traumatic injuries and had a brain MRI. Case reports, original research, and systematic reviews were included. The citation was excluded when (1) the abstract or title indicated non-trauma patients, (2) a trauma patient had a major neurologic deficit immediately following injury, or (3) the article was published in a non-English language. The authors sought to exclude traumatic brain injury patients and focus only on trauma patients without brain injury, who had an acute mental status deterioration consistent with CFES with an associated MRI brain. Full-text articles were reviewed for the 30 included citations. Twenty full-text articles were found that described trauma patients with CFES who had an associated brain MRI but were without a major neurologic deficit immediately following injury. A review of the 20 full-text article bibliographies identified an additional four relevant manuscripts. Figure 1 displays a flowchart of article selection for the literature review.

Assessments for NCWMH, Pre-CFES Cognition, Neurologic Deterioration, and Skeletal Fracture Detection

NCWMH was delineated by adapting Parizel’s description of multiple nodular or punctate foci of high signal intensity in the white matter on T2WI/FLAIR or scattered bright spots in a dark background on DWI [7]. These increased signal densities were also described as vasogenic edema and cytotoxic edema. If those image findings were clearly identified by either provided images or by description, they were recorded. Ambiguous imaging findings were not included. Patients were included in the current review if the manuscript provided MRI pictorials or if there was an adequate description of the MRI to determine the presence or absence of NCWMH. If there was a disagreement on the patients, the authors met to determine if the patient should be included. When an adequate description was available in the manuscript, the pre-CFES cognitive status was documented as Glasgow Coma Scale (GCS) score = 14-15 (yes or no). When a sufficient description was available in the manuscript, the degree of neurologic deterioration with CFES was recorded as coma or GCS ≤ 8 (yes or no). When documented in the manuscript, the presence of a skeletal fracture was documented (yes or no).

Results

Author CFES Case Reports

**Figure 2:**
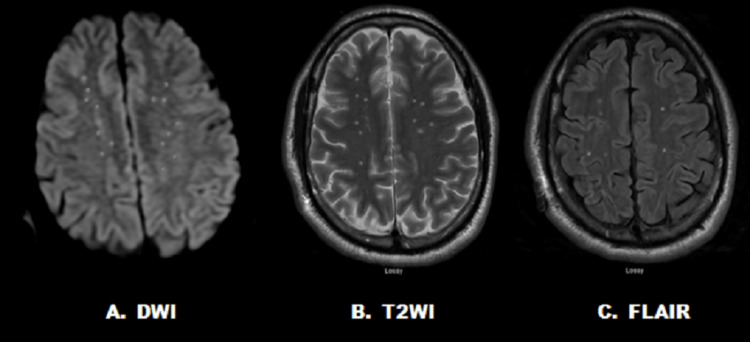
Case 1 with numerous cerebral white matter hyperintensities on (A) DWI, (B) T2WI, and (C) FLAIR MRI sequences within five days of traumatic injury DWI, diffusion-weighted image; T2WI, T2-weighted image; FLAIR, fluid-attenuated inversion recovery; MRI, magnetic resonance imagining.

Case report 1: A 48-year-old male presented with GCS 15 after a motorcycle crash. Injuries included a left displaced tibia-fibula fracture, left-sided rib fractures, and grade 1 splenic laceration. The patient underwent open reduction internal fixation of the displaced long bone fractures within 24 hours of injury. During the overnight period, he developed increased agitation and confusion, and his GCS decreased to 14. A head computed tomography (CT) image was negative for any acute findings. He underwent an echocardiogram with saline contrast that showed normal structure and was negative for atrial septal defect and patent foramen ovale. He had normal left ventricle, systolic, and diastolic function with an ejection fraction of 55-60%. He had ongoing confusion and increased respiratory requirements. On postoperative day four, he underwent a brain MRI with T2WI, DWI, and FLAIR sequences, which showed hyperintense areas throughout the bilateral corona radiata, bilateral thalamus, bilateral basal caudate head, and white matter consistent with acute infarction (Figure 2). These MRI findings were consistent with the diagnosis of CFES. By postoperative day eight, the patient’s mental status improved with supportive care, and on postoperative day 14, he was discharged with GCS 15 to a rehabilitation facility.

**Figure 3:**
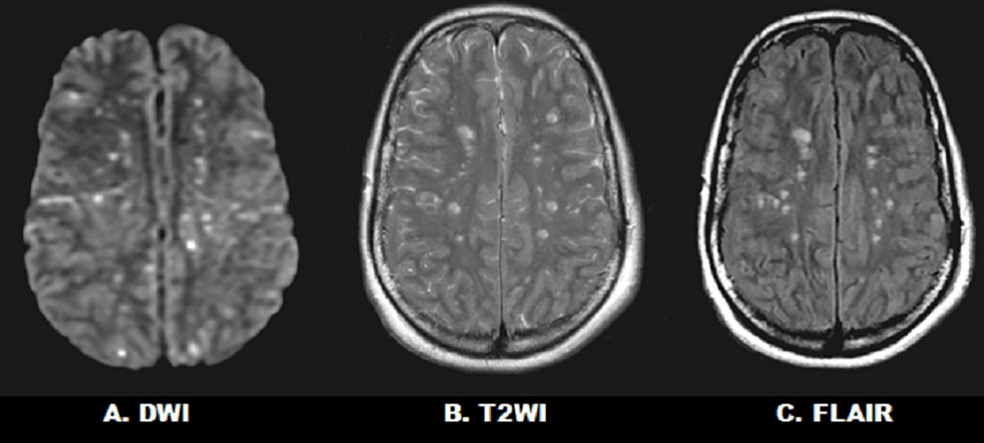
Case 2 with numerous cerebral white matter hyperintensities on (A) DWI, (B) T2WI, and (C) FLAIR MRI sequences within 48 hours of traumatic injury DWI, diffusion-weighted image; T2WI, T2-weighted image; FLAIR, fluid-attenuated inversion recovery; MRI, magnetic resonance imagining.

Case report 2: A 43-year-old female presented to the trauma service after a motor vehicle crash. The patient had a GCS 15, multiple rib fractures, right calcaneus and right medial malleolus fractures, and left intra-articular distal radius fracture. She underwent fixation of her orthopedic injuries on the day of arrival. Overnight, after orthopedic surgery, the patient developed altered mental status with a deterioration to GCS 8 requiring tracheal intubation. A head CT and CT angiogram of the neck were both unremarkable. A brain MRI was performed and included T2WI, DWI, and FLAIR sequences. DWI showed innumerable relatively symmetric bilateral punctate hyperintense foci of periventricular, subcortical, and deep areas of cerebral white matter restricted diffusion. T2WI and FLAIR demonstrated susceptibility artifacts and numerous cerebral white matter hyperintensities (Figure 3). She underwent a transesophageal echocardiogram that was negative for atrial septal defect and patent foramen ovale. She had normal systolic function with an ejection fraction of 55-60%. She had no atrial thrombus. The patient underwent intracranial pressure monitor placement initially, and subsequent tracheostomy and feeding tube placement. She was later discharged with GCS 10T (eyes 4, motor 5, verbal 1 tracheostomy) to a long-term care facility. She continued to improve, was advanced to an oral diet, and had her tracheostomy decannulated. After one month at the long-term care facility, she was subsequently transferred to acute rehab with GCS 15.

CFES Literature Review and Two Case Reports

There were 24 publications reviewed with a total of 131 patients [8-31], and our two cases increased the total to 133 patients. The patients from the reviewed manuscripts were included only after an independent review by each author based on the inclusion criteria described in the methods.

Most included publications were case reports (21 publications) and three publications had relatively large datasets (patient numbers 24-39) [11,27,28]. When the MRI sequence was stipulated in the manuscript for detecting NCWMH (63 patients), the total number of patients in this review by sequence was DWI in 24, T2WI in 57, and FLAIR in 10 patients (Table 1). Analysis of the MRI showed that the majority of CFES patients had NCWMH (91.6%). The extracted data are summarized in Table 1. The proportion of CFES patients with NCWMH was similar in the larger studies (88.7%, 86/97) when compared to the case reports (100%, 34/34; P = 0.0655). Almost half of the study patients had admission GCS documented, and the vast majority did not present with an altered level of consciousness (Table 2). When described, virtually all the patients had evidence of neurological deterioration during the hospital course that led to the workup of CFES with MRI (Table 3). Half of the patients manifest with coma or GCS ≤ 8 (Table 3). All but one of the patients had a documented fracture prior to CFES onset (Table 4). The suspected etiology of CFES in this one patient was not further described [28].

**Table 1:** Proportion of CFES patients with NCWMH CFES, cerebral fat embolism syndrome; CI, confidence interval; MRI, magnetic resonance imaging; NCWMH, numerous cerebral white matter hyperintensities; DWI, diffusion-weighted image; T2WI, T2-weighted image; FLAIR, fluid-attenuated inversion recovery.

Study	All patients	Patients with NCWMH	MRI sequence described
Case report 1, 2023	1	1	DWI/T2WI/FLAIR
Case report 2, 2023	1	1	DWI/T2WI/FLAIR
Aggarwal, 2019 [8]	1	1	T2WI
Ajayakumar, 2022 [9]	1	1	DWI/T2WI
Algahtani, 2023 [10]	3	3	T2WI/FLAIR
Armstrong, 2022 [11]	34	34	T2WI
Chase, 2020 [12]	3	3	Not described
Davis, 2020 [13]	7	7	DWI/T2WI
Gearhart, 2023 [14]	1	1	DWI
Hoiland, 2021 [15]	2	2	DWI/T2WI
Hsu, 2021 [16]	1	1	DWI
Huang, 2019 [17]	1	1	DWI
Huang, 2021 [18]	1	1	DWI/T2WI/FLAIR
In, 2023 [19]	1	1	DWI
Josan, 2021 [20]	1	1	DWI/T2WI/FLAIR
Kanda, 2023 [21]	3	3	Not described
Kassimi, 2022 [22]	1	1	DWI/T2WI
Mburu, 2022 [23]	1	1	DWI/T2WI/FLAIR
Ostlie, 2022 [24]	1	1	DWI/T2WI
Qin, 2023 [25]	1	1	Not described
Saran, 2020 [26]	1	1	DWI
Shaikh, 2018 [27]	24	23	Not described
Singh, 2022 [28]	39	29	Not described
Tsuru, 2020 [29]	1	1	DWI
Wang, 2021 [30]	1	1	T2WI/FLAIR
Zhang, 2022 [31]	1	1	DWI/T2WI/FLAIR
Total	133	122 (91.7%)	95% CI: 85.6-95.2%

**Table 2:** Pre-CFES GCS 14-15 * 95% confidence interval = 84.6%-97.5%; CFES, cerebral fat embolism syndrome; GCS, Glasgow Coma Scale score.

Study	All patients	Patients with GCS 14-15
Case report 1, 2023	1	1
Case report 2, 2023	1	1
Ajayakumar, 2022 [9]	1	1
Algahtani, 2023 [10]	2	2
Armstrong, 2022 [11]	34	31
Chase, 2020 [12]	3	2
Davis, 2020 [13]	7	7
Gearhart, 2023 [14]	1	1
Hoiland, 2021 [15]	2	2
Hsu, 2021 [16]	1	1
Huang, 2019 [17]	1	1
In, 2023 [19]	1	1
Josan, 2021 [20]	1	1
Kassimi, 2022 [22]	1	1
Mburu, 2022 [23]	1	1
Ostlie, 2022 [24]	1	1
Qin, 2023 [25]	1	1
Tsuru, 2020 [29]	1	1
Wang, 2021 [30]	1	1
Total	62	58 (93.5%*)

**Table 3:** Neurologic deterioration with CFES CFES, cerebral fat embolism syndrome; ND, neurologic deficit; Major ND, neurologic deterioration with onset of coma or Glasgow Coma Scale score ≤ 8.

Study	All patients	Patients with ND	Patients with major ND
Case report 1, 2023	1	1	0
Case report 2, 2023	1	1	1
Ajayakumar, 2022 [9]	1	1	1
Algahtani, 2023 [10]	3	3	3
Armstrong, 2022 [11]	34	31	2
Chase, 2020 [12]	3	3	2
Davis, 2020 [13]	7	7	Unknown
Gearhart, 2023 [14]	1	1	1
Hoiland, 2021 [15]	2	2	2
Hsu, 2021 [16]	1	1	0
Huang, 2019 [17]	1	1	0
Huang, 2021 [18]	1	1	0
In, 2023 [19]	1	1	1
Josan, 2021 [20]	1	1	0
Kassimi, 2022 [22]	1	1	1
Mburu, 2022 [23]	1	1	0
Ostlie, 2022 [24]	1	1	1
Qin, 2023 [25]	1	1	1
Saran, 2020 [26]	1	1	1
Shaikh, 2018 [27]	23	23	13
Singh, 2022 [28]	39	39	29
Tsuru, 2020 [29]	1	1	1
Wang, 2021 [30]	1	1	1
Zhang, 2022 [31]	1	1	1
Total	128	125 (97.7%)	62/118 (52.5%)

**Table 4:** Presence or absence of fracture in CFES patients CFES, cerebral fat embolism syndrome.

Study	All patients	Patients with fracture
Case report 1, 2023	1	1
Case report 2, 2023	1	1
Aggarwal, 2019 [8]	1	1
Ajayakumar, 2022 [9]	1	1
Algahtani, 2023 [10]	3	3
Armstrong, 2022 [11]	27	27
Chase, 2020 [12]	3	3
Davis, 2020 [13]	7	7
Gearhart, 2023 [14]	1	1
Hoiland, 2021 [15]	2	2
Hsu, 2021 [16]	1	1
Huang, 2019 [17]	1	1
Huang, 2021 [18]	1	1
In, 2023 [19]	1	1
Josan, 2021 [20]	1	1
Kanda, 2023 [21]	3	3
Kassimi, 2022 [22]	1	1
Mburu, 2022 [23]	1	1
Ostlie, 2022 [24]	1	1
Qin, 2023 [25]	1	1
Saran, 2020 [26]	1	1
Singh, 2022 [28]	39	38
Tsuru, 2020 [29]	1	1
Wang, 2021 [30]	1	1
Zhang, 2022 [31]	1	1
Total	102	101

## Discussion

The exact mechanism of CFES is not known. Multiple theories have been proposed as to the etiology of CFES, including the “Mechanical theory” and the “Biochemical theory.” The “Mechanical theory” postulates that external forces cause fat droplets to flow into the venous system, lodge into pulmonary microvasculature, and then into the arterial system via right to left shunts [2]. Neither of our two cases had evidence of right-to-left shunts. The “Biochemical theory” proposes that fat globules enter the pulmonary vasculature and are broken down into free fatty acids, which leads to a microinflammatory response [2]. At the brain level, the fat droplets cause local ischemia and inflammation [2]. The authors postulate that the fat emboli cause local ischemia and the release of inflammatory mediators, which lead to neurological changes.

Brain MRI findings in CFES patients are diverse and include hypointensities with microbleeds and hyperintensities with ischemia-edema that involve several different nervous system anatomic structures [5,6]. Further, hyperintensities may appear as numerous foci or confluent pathology [5,6]. CFES is a rare clinical entity that often begins with neurologic deterioration after hospital admission that ranges from mild (GCS 14) to serious with a coma or GCS ≤ 8. Half of the patients in our systematic literature review had major neurological deterioration. Identifying a common MRI imaging pattern would likely be helpful from a clinical standpoint. The two CFES cases we reported occurred among a group of 1,019 (0.2%) fracture patients admitted to our trauma center during the 2022 calendar year. These patients had neurological decompensation after the fixation of skeletal injuries, which led to investigative MRI imaging. The images had the classic NCWMH finding. We hypothesized that NCWMH would be a common MRI finding in CFES patients reported in the recent literature. NCWMH was discovered to be present in over 90% of patients in the current systematic review. In addition to finding NCWMH manifest on DWI, numerous examples of this pattern appeared on T2WI and FLAIR sequences. Because ischemic infarction is known to create a hyperintense lesion on DWI, T2WI, and FLAIR [32], we speculate that the NCWMH likely represent ischemic changes. DWI has been found to be the most sensitive method for detecting cerebral ischemia in the hyperacute state [33].

The overlap in imaging findings between diffuse axonal injury (DAI) and CFES with NCWMH may present a diagnostic dilemma. The lesions seen on MRI are not unique to CFES [34] and can also be seen with DAI [35]. DAI presents with a variety of MRI findings, which can include hypointense microbleeds as well as hyperintensities (NCWMH). Gradient recalled echo (GRE) T2WI may be better at detecting microbleeds than conventional T2WI [36]. NCWMH can also appear in other causes of microembolism, such as chronic small vessel ischemic disease in older adults. However, chronic small vessel disease is commonly seen on head CT, whereas the NCWMH MRI pattern in acute CFES is not typically seen on CT.

Although there may be difficulty in discerning CFES and DAI patients based on MRI scans, early postinjury neurological status will likely help to differentiate the clinical conditions. Moe et al. reviewed 490 traumatic brain injury patients who underwent an MRI of the brain [37]. Of the 282 with DAI, only 3.9% had an admission GCS score of 14-15 [37]. Patients with near-normal GCS on admission are highly unlikely to have DAI. Our data showed that CFES was associated with near-normal consciousness prior to the onset of CFES in over 90% of patients. In the current review, virtually all CFES patients had an in-hospital neurological deterioration. Future studies comparing MRI findings between CFES and DAI would be insightful.

## Conclusions

Our study is the most recent to date to examine CFES in trauma patients. Orthopedic fractures and evidence of in-hospital neurological deterioration are seen with CFES. Numerous hyperintensities seen in the cerebral white matter of the brain MRI (T2WI and DWI) are common patterns seen in CFES. The presence of NCWMH on a DWI, T2WI, or FLAIR MRI sequence in patients with a neurologic deterioration and the presence of fat embolism syndrome or a skeletal fracture is consistent with multiple ischemia foci and CFES. Recognizing neurological deterioration in trauma patients with orthopedic fractures, without an initial traumatic brain injury, and subsequent MRI brain findings of NCWMH are key attributes in the diagnosis of CFES.
